# Synopsis of the genus *Ulomorpha* Osten Sacken, 1869 (Diptera, Limoniidae) in Japan

**DOI:** 10.3897/zookeys.999.52831

**Published:** 2020-11-30

**Authors:** Daichi Kato, Kozo Watanabe, Levente-Péter Kolcsár

**Affiliations:** 1 Echigo-Matsunoyama Museum of Natural Sciences, ‘Kyororo’, 1712-2 Matsunoyama, Tôkamachi, 942-1411, Japan Echigo-Matsunoyama Museum of Natural Sciences Tôkamachi Japan; 2 Center for Marine Environmental Studies (CMES), Ehime University, Matsuyama, Ehime 790-8577, Japan Ehime University Ehime Japan

**Keywords:** Crane flies, male terminalia, new species, taxonomy, Tipuloidea

## Abstract

Japanese species of the genus *Ulomorpha* Osten Sacken, 1869 are revised and *U. amamiana* Kato & Kolcsár, **sp. nov.** and *U. longipenis* Kato & Kolcsár, **sp. nov.** are described. A key to the four Japanese species of the genus is provided, with images of habitus and wings, and drawings of their male terminalia. *Ulomorpha amamiana* Kato & Kolcsár, **sp. nov.** is the first representative of the genus discovered from the Oriental region.

## Introduction

*Ulomorpha* Osten Sacken, 1869 is a small genus of the subfamily Limnophilinae and so far includes two Palaearctic and eight Nearctic species (Oosterbroek 2020). The adults are characterized by having conspicuous macrotrichiae on wing cells and cell R_3_ sessile to subsessile. A similar condition is present in the limnophiline genera *Adelphomyia* Bergroth, 1891, *Paradelphomyia* Alexander, 1936, and Limnophila (Lasiomastix) Osten Sacken, 1860 but only in *Ulomorpha* are macrotrichiae proximal to the cord present. Morphological analyses of the characters of immature stages demonstrate that *Ulomorpha* is closely related to *Pilaria* Sintenis, 1889 (Oosterbroek and Theowald 1991). Cladistic analysis of the adult morphological characters recovered a close relationship of *Ulomorpha* with *Pseudolimnophila* Alexander, 1919, *Pilaria*, and *Hexatoma* Latreille, 1809 owing to the bifid interbase in males (Ribeiro 2008).

Immature stages of *U. pilosella* (Osten Sacken, 1860) were described by Alexander (1920a) and were reported from soil rich in organic matter in shaded woods (Alexander and McAtee 1920), but the biology of the genus is otherwise poorly known.

Two species of the genus have been recorded from Japan, *U. nigricolor* Alexander, 1924 (Honshu, Shikoku, and Kyushu islands) and *U. polytricha* Alexander, 1930 (Yakushima Island) (Nakamura 2014; Oosterbroek 2020). In this study, the Japanese species of the genus are revised, and two new species are described, with additional faunistic records including the first representative of the genus from the Oriental region. Images of wings and habitus, drawings of male terminalia, and a key to the Japanese species are provided.

## Materials and methods

**Figure 1.**
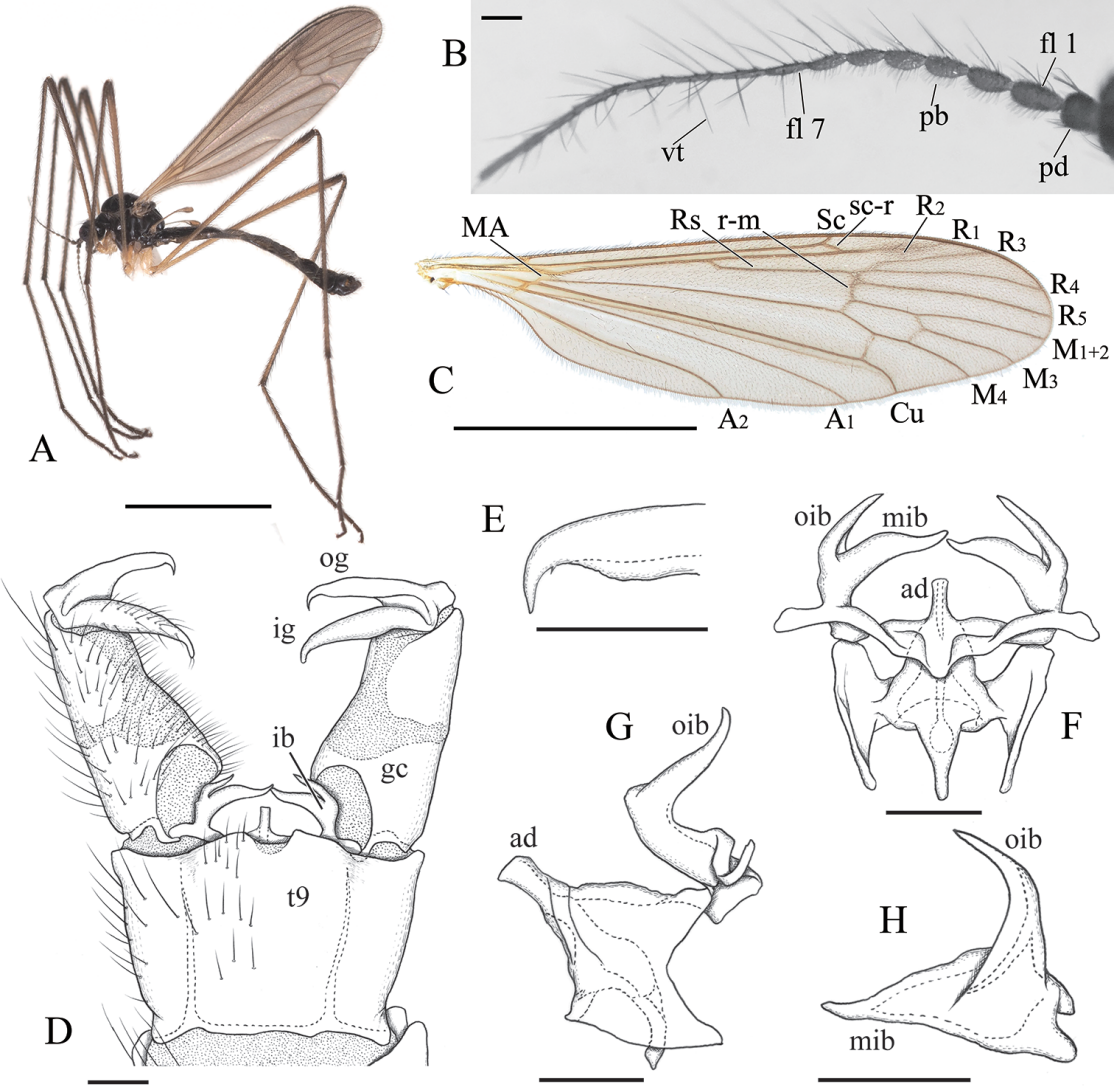
*Ulomorpha amamiana* Kato & Kolcsár, sp. nov. **A** habitus, male **B** basal part of antenna, lateral view **C** wing **D** male terminalia, dorsal view **E** tip of outer gonostylus, dorsal view **F** aedeagal complex, dorsal view **G** aedeagal complex, lateral view **H** interbase, posterodorsal view. Abbreviations: ad – aedeagus; fl – flagellomere; ib – interbase; ig – inner gonostylus; gc – gonocoxite; mib – medial lobe of interbase; og – outer gonostylus; oib – outer lobe of interbase; pb – pubescence; pd – pedicel; t9 – tergite 9; vt – verticil. Scale bars: 3 mm (**A, C**); 0.1 mm (**B, D, E–H**).

The specimens were collected by insect nets by D. Kato and L.-P. Kolcsár and either preserved in 90% ethanol or pinned. Overall descriptions of the species were based on the observations made through a Leica MZ7.5 stereomicroscope. Male terminalia of pinned specimens were heated in a solution of 10% KOH for several minutes, then rinsed in a solution of 70% ethanol with 3% acetic acid for neutralization and transferred to glycerol for examination and drawing. The treated genitalia were preserved in genitalia tubes filled with glycerol and the tubes were pinned below the body remains. Drawings were made using the stereomicroscope equipped with a grid eyepiece micrometer. Habitus and wings were photographed with an Olympus OM-D E-M5 Mark II using a M. Zuiko Digital ED 60 mm F2.8 macro lens. Wing venation terminology follows the traditional system, based on McAlpine (1981) and Merz and Haenni (2000), with a modifications from Starý (2008); CuA is referred to here as Cu (Fig. 1C). General distributions of species are from the Catalogue of the Craneflies of the World (Oosterbroek 2020).

### Depositories

**BLKU**Biosystematic Laboratory, Kyushu University, Japan;

**CKLP** Private Collection of L.-P. Kolcsár;

**USNM**National Museum of Natural History, Smithsonian Institution, Washington, DC, USA.

## Taxonomic treatment

### Ulomorpha

Taxon classificationAnimaliaDipteraLimoniidae

Osten Sacken, 1869

65F969F2-C433-50D5-81AC-5409BDFD0289

**Figure 2.**
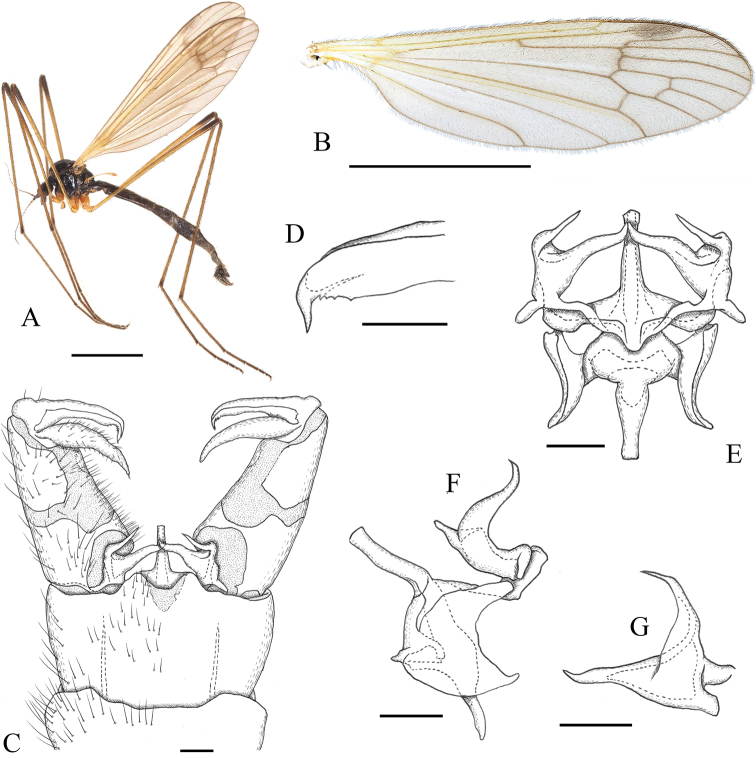
*Ulomorpha longipenis* Kato & Kolcsár, sp. nov. **A** habitus, male **B** wing **C** male terminalia, dorsal view **D** tip of outer gonostylus, dorsal view **E** aedeagal complex, dorsal view **F** aedeagal complex, lateral view **G** interbase, posterodorsal view. Scale bars: 3 mm (**A, B**); 0.1 mm (**C–G**).

**Figure 3.**
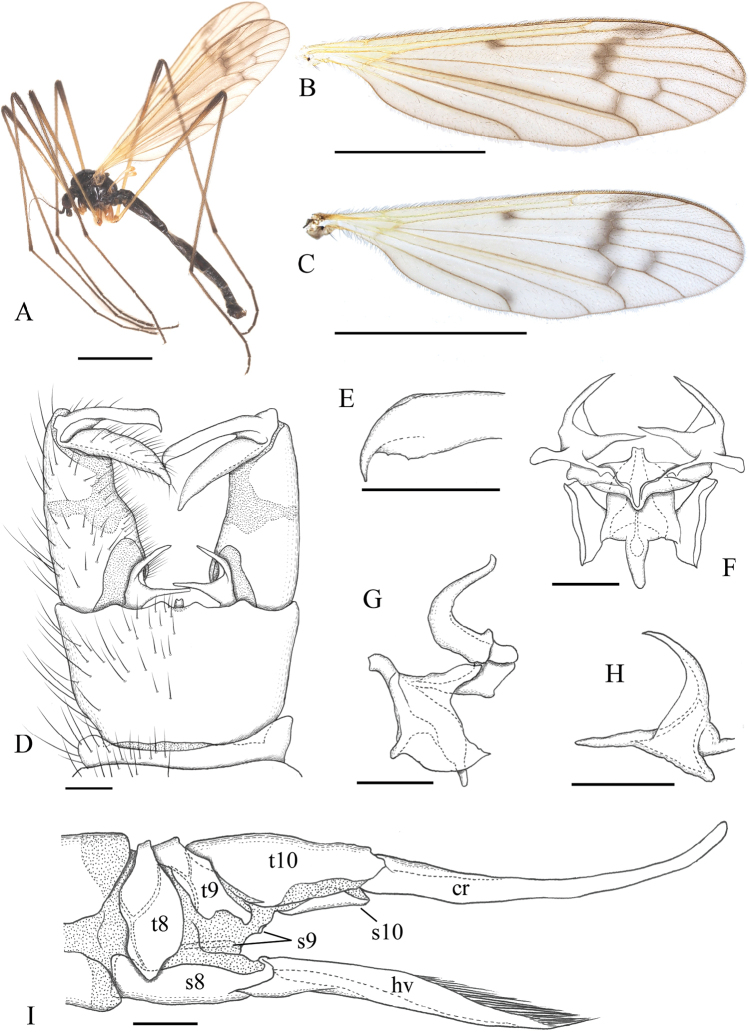
*Ulomorpha nigricolor* Alexander, 1924. **A** habitus, male **B, C** wing **D** male genitalia, dorsal view **E** tip of outer gonostylus, dorsal surface **F** aedeagal complex, dorsal view **G** aedeagal complex, lateral view **H** interbase, posterodorsal view **I** female ovipositor, lateral view. Abbreviations: cr – cercus; hv – hypogynial valve; s – sternite; t – tergite. Scale bars: 3 mm (**A–C**); 0.1 mm (**D–H**); 0.3 mm (**I**).

**Figure 4.**
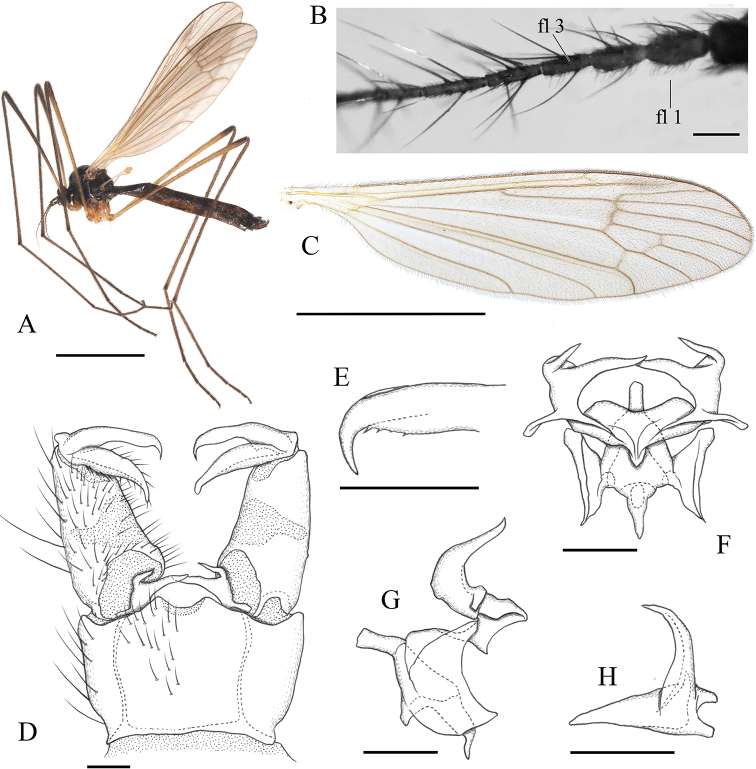
*Ulomorpha polytricha* Alexander, 1930. **A** habitus, male **B** basal part of antenna, lateral view **C** wing **D** male genitalia, dorsal view **E** tip of outer gonostylus, dorsal surface **F** aedeagal complex, dorsal view **G** aedeagal complex, lateral view **H** interbase, posterodorsal view. Scale bars: 3 mm (**A, C**); 0.1 mm (**B, D–H**).

**Figure 5.**
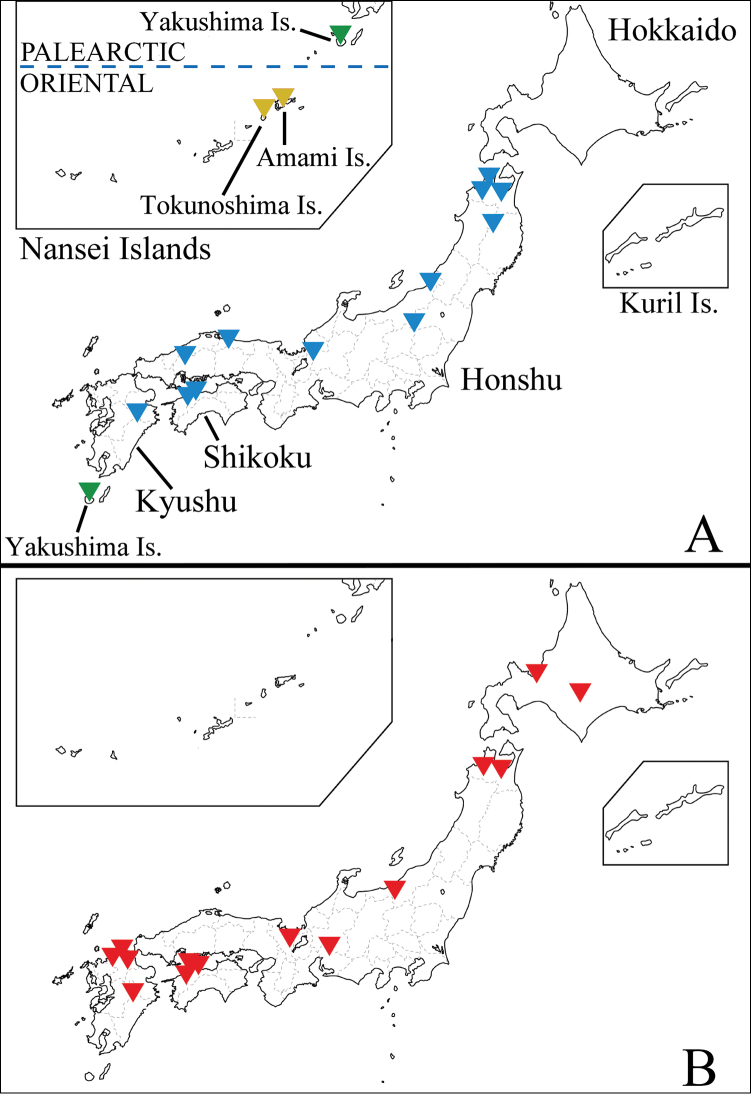
Distribution map of Japanese species of *Ulomorpha*. **A***U. amamiana* Kato & Kolcsár, sp. nov. (orange), *U. nigricolor* Alexander, 1924 (blue), *U. polytricha* Alexander, 1930 (green) **B***U. longipenis* Kato & Kolcsár, sp. nov. (red).

Figs 1 , 2 , 3 , 4 , 5

#### Type species.

*Limnophila pilosella* Osten Sacken, 1860 by original designation (Osten Sacken 1869: 232).

#### General description of Japanese species of *Ulomorpha* Osten Sacken, 1869.

General coloration shiny black, mainly yellow on legs, body clothed with relatively long setae.

***Head*** with eye dichoptic, separated by about twice width of scape on dorsal part and about 1.5 times on ventral part; rostrum 2/3–3/4 length of scape; antenna 16–segmented, 3–4 times as long as head; scape cylindrical, about twice length of pedicel and as wide as pedicel; pedicel globular; flagellum with verticils, longer on middle segments, at most 2.5 times as long as each segment, shortest on apical segment, shorter than apical segment; basal flagellomeres (Figs 1B, 4B) long-oval with pubescences ventrally, as long as 1/2–1 width of each segment; distal flagellomeres long and cylindrical; palpus 5-segmented, shortest on basal segment and longest on apical segment.

***Thorax*** with prescutal pit roundish; tuberculate pit situated near anterior margin on prescutum; meron small, largely membranous on posterior part, separating mid and hind coxae by about 1/2 width of coxa; wing (Figs 1C, 2B, 3B, C, 4C) covered with macrotrichiae except basal part; Sc end at level of distal 1/3 to tip of Rs; crossvein sc-r near tip of Sc; MA present; Rs origin near middle between MA and distal end of Rs; crossvein R_2_ indistinct, situated at basal 1/2–1/4 of R_3_; R_3+4_ very short, often absent; M_1+2_ not forked (forked in some Nearctic species); cell d closed; crossvein m-cu situated near middle of cell d; Cu curved posteriorly near wing margin; halter about half length of hind coxa; legs with tibial spurs 1 + 2 + 2; tarsomeres 1 to 3 each with 1 tarsal spur; claw about half as long as tarsomere 5, covered with small, hair-like setae on basal half, ventral margin smooth, without teeth; arolium present.

***Male terminalia*** (Figs 1D–H, 2C–G, 3D–H, 4D–H) with segment 9 ring-shaped; tergite 9 and sternite 9 fused laterally; tergite 9 with pair of small roundish lobes at posterior margin; sternite 9 widely concaved at middle of posterior margin; gonocoxite about 1.5 times as long as tergite 9, slightly wide on basal part; gonostyli 1/2–2/3 length of gonocoxite; outer gonostylus black, wide on basal part, tip narrowed into small curved spine; inner gonostylus slightly longer than outer gonostylus, gradually narrowed to tip; interbase bilobed distally, outer lobe narrow and directed posterodorsally, acute at tip; medial lobe larger and directed medially, narrowed to tip; each base of interbase extending medially and fused into bridge.

***Ovipositor*** (Fig. 3I) long, more than 1/3 as long as abdomen; cercus more than 1.5 times as long as tergite 10, weakly upcurved on distal part; hypogynial valve more than 1.5 times as long as sternite 8, almost straight, tip ending at near level of middle of cercus.

### Key to Japanese species of *Ulomorpha* Osten Sacken, 1869

**Table:** 

1	Wing with distinct dark spot at origin of Rs and with distinct dark seam on fork of Rs to crossvein m-cu (Fig. 3B, C); prescutum shiny with narrow longitudinal line of pruinosity at center	***U. nigricolor* Alexander, 1924**
–	Wing without dark spot at origin of Rs and conspicuous dark seam on crossvein m-cu (Figs 1C, 2B, 4C); prescutum sparsely covered with pruinosity or entirely shiny	**2**
2	Wing with stigma distinctly dark and fork of Rs to crossvein r-m weakly dark (Fig. 2B); aedeagus with rod-shaped part more than three times as long as wide (Fig. 2F)	***U. longipenis* Kato & Kolcsár, sp. nov.**
–	Wing with stigma and dark seam on fork of Rs to crossvein m-cu indistinct (Figs 1C, 4C); aedeagus with rod-shaped part twice as long as wide (Figs 1G, 4G)	**3**
3	Male flagellum oval on basal 4 segments, with pubescence on basal 6–7 segments ventrally (Fig. 1B); medial lobe of interbase medial to base of outer lobe about 1.5 times as long as wide (Fig. 1H)	***U. amamiana* Kato & Kolcsár, sp. nov.**
–	Male flagellum oval on basal 2–3 segments, with pubescence on basal 2–3 segments ventrally (Fig. 4B); medial lobe of interbase medial to base of outer lobe more than twice as long as wide (Fig. 4H)	***U. polytricha* Alexander, 1930**

### Ulomorpha amamiana

Taxon classificationAnimaliaDipteraLimoniidae

Kato & Kolcsár sp. nov.

F069026E-89C5-581D-93AD-7784CB5FF2CB

http://zoobank.org/7CB700D7-FBCE-43C9-817D-D91229A29886

Figs 1 , 5A

#### Material examined.

***Holotype*** ♂, pinned. Original label: “JAPAN, Nansei Islands, Amami I., Yamato-son, Yuwangama; alt. 250 m; 28°21.07'N, 129°25.31'E; 31 Mar. 2019; D. Kato leg.” “HOLOTYPE *Ulomorpha amamiana* Kato & Kolcsár, sp. nov. [red label]” (BLKU).

***Paratypes*.** Japan: [Nansei Islands] Amami I.: • 2♂, 1♀; same data as holotype • 1♀; Setouchi-chô, Shinokawa, Yakugachi-gawa River; alt. 130 m; 28°13.25'N, 129°18.88'E; 3 Apr. 2019; D. Kato leg. (BLKU). Tokunoshima I.: • 2♂, 1♀; Amagi-chô, Tôbe, Mt Minada-yama; alt. 300 m; 27°47.72'N, 128°56.22'E; 2 Apr. 2019; D. Kato leg. (BLKU) • 2♂; Tokunoshima-chô, Mt Inokawa-dake to Mt Hage-dake; 27°45.89'N, 128°59.5'E; 30 Sep. 2013; D. Kato leg. (BLKU) • 2♂, 2♀; Tokunoshima-chȏ, Todoroki, near Mt Sasontsuji-dake; alt. 200 m; 27°50.36'N, 128°56.45'E; 2 Apr. 2019; D. Kato leg. (BLKU).

#### Diagnosis.

Body blackish. Vertex and scutum sparsely pruinose. Flagellomeres oval to bacilliform on basal 4 segments; ventral sides with pubescences on basal 6–7 segments. Wing brownish tinged, unpatterned; stigma sometimes indistinctly darker. Halter yellow. Interbase with outer lobe shorter than medial lobe in dorsal view; medial lobe medial to base of outer lobe about 1.5 times as long as wide. Aedeagus with rod-shaped distal part, twice as long as wide and almost straight.

#### Description.

**Male**. Body length 5.6–8.0 mm, wing length 5.8–7.9 mm.

***Head*:** subnitidous black, sparsely dusted with gray pruinosity; vertex with brighter gray pruinosity at anterior end. Rostrum and mouthparts brown to dark brown. Antenna brown to dark brown; scape and pedicel sometimes slightly darker; basal 4 flagellomeres oval to bacilliform; basal 6–7 segments covered with pubescences ventrally (Fig. 1B).

***Thorax*:** subnitidous dark brown to black, sparsely dusted with brownish pruinosity; postpronotum yellowish or brownish. Wing (Fig. 1C) tinged with brown; basal region yellowish; stigma absent or indistinctly darker; veins dark brown, yellowish on basal part of wing; barely dark seam sometimes present on fork of Rs to crossvein r-m. Halter yellow. Legs mainly yellow to dusky yellow; fore coxa sometimes weakly dark on basal part; femora narrowly dark at tips; dark area on fore femur sometimes weak and occupying distal half; tibiae narrowly dark at tips; tarsi weakly dark from tip of tarsomere 1 to apical segment.

***Abdomen*:** subnitidous dark brown to black, sparsely covered with brownish pruinosity.

***Male terminalia*** (Fig. 1D–H): caudal margin of tergite 9 roundly produced at middle, with small U-shaped notch at center. Outer gonostylus in dorsal view (Fig. 1E) with tip narrowed and curved anteriorly; concaved margin with indistinct teeth. Interbase with outer lobe shorter than medial lobe in dorsal view (Fig. 1F); outer lobe wide at base, inner basal end situated near middle of medial lobe (Fig. 1H); medial lobe strongly narrowed distally, distal part medial to base of outer lobe about 1.5 times as long as wide (Fig. 1H); aedeagus with rod-shaped distal part, twice as long as wide andalmost straight (Fig. 1F, G).

**Female**. Body length 7.0–9.2 mm, wing length 5.8–8.0 mm.

Generally resembling male. Antenna with flagellum oval only on segment 1, only basal 2 flagellomeres less distinctly covered with pubescences.

***Ovipositor*:** dark brown; yellow on cercus hypogynial valve, and distal 1/3 of tergite 10; cercus 2.0–2.5 times as long as tergite 10.

#### Etymology.

The name of this species is derived from that of the type locality, Amami Island. The name is deemed to be a latinized adjective in nominative singular.

#### Distribution.

Japan (Nansei Islands: Amami Islands (Amami and Tokunoshima Island)) (Fig. 5A).

#### Biogeographic notes.

The crane fly fauna of the Nansei Islands or Ryukyu Arc is poorly known, and the new species and new distribution records are recently reported (Kato 2020; Kolcsár et al. 2020, in press). The Amami Islands are in the northeastern part of the Oriental faunal realm. The hypothetical boundary between the Palearctic and Oriental (Indomalaya) realms, the Watase line or Tokara gap, is delimited between Yakushima/Tanegashima and Amami islands (Komaki and Igawa 2017). The Ryukyu Islands arc once formed a continental margin arc which connected to the eastern margin of the Asian continent and served as an important land bridge (Osozawa et al. 2012). The presence of *Ulomorpha* in the Amami Islands is not surprising, as the group occurs in Yakushima Island. However, *U. amamiana* Kato & Kolcsár, sp. nov. is the first representative of the genus in the Oriental faunal realm. Future phylogenetic analyses may help understanding of the biogeography of the group in the area.

#### Remarks.

This species is similar to *U. polytricha* Alexander, 1930. See the key to the Japanese species above for differentiation and diagnostic characters.

### Ulomorpha longipenis

Taxon classificationAnimaliaDipteraLimoniidae

Kato & Kolcsár sp. nov.

879907CF-BA19-5DBC-9AAD-E049FB0FB18E

http://zoobank.org/E2EA56ED-B89A-43B8-B668-03E0E4E9FDAC

Figs 2 , 5B

#### Material examined.

***Holotype*** ♂, pinned. Original label: “JAPAN, Hokkaido, Sapporo-shi, Minami-ku, Moiwashita, Mt Moiwa-yama; alt. 200 m; 43°0.84'N, 141°20.01'E; 23 Jun. 2014; D. Kato leg.” “HOLOTYPE *Ulomorpha longipenis* Kato & Kolcsár, sp. nov. [red label]” (BLKU).

***Paratypes*.** Japan, [Hokkaido] • 3♂; same data as holotype • 1♀; Hidaka-chô, Chisaka, tributary of Saru-gawa River; alt. 739 m; 42°58.39'N, 142°40.79'E; 28 Jul. 2019; L.-P. Kolcsár leg. (CKLP). [Honshu] • 1♀; Aomori, Nishimeya-mura, Kawaratai, Ôkawa Path; alt. 300 m; 40°30.04'N, 140°12.24'E; 30 Jun. 2013; D. Kato leg. (BLKU) • 3♂; Aomori, Towada-shi, Okuse, Tsutanuma Path; alt. 460 m; 40°35.45'N, 140°57.42'E; 1 Jun. 2014; D. Kato leg. (BLKU) • 1♂; same data as previous except 21 Jun. 2014 • 1♂; Niigata, Tôkamachi-shi, Matsunoyama-Amamizukoshi, Mt Amamizu-yama; alt. 920 m; 37°1.46'N, 138°33.77'E; 2 Aug. 2019; D. Kato leg. (BLKU) • 2♂; Aichi, Seto-shi, Iwaya-chô, near Iwayadô Park; alt. 300 m; 35°14.37'N, 137°9.05'E; 4 May 2016; D. Kato leg. (BLKU) • 1♀; Kyoto, Kibune; 35°7.29'N, 135°45.45'E (rough coordinate, altitude unknown); ?.IV.1935; M. Tokunaga leg. (USNM). [Shikoku] • 1♂, 1♀; Ehime, Kumakôgen-chô, Chichinokawa; alt. 580 m; 33°36.29'N, 132°51.35'E; 19 May 2019; L.-P. Kolcsár leg. (CKLP) • 1♂; Ehime, Kumakôgen-chô, Hinoura; alt. 722 m; 33°35.13'N, 132°57.71'E; 19 May 2019; L.-P. Kolcsár leg. (CKLP) • 2♂; Ehime, Kumakôgen-chô, Nishidani, near Prefectural road 328; alt. 1430 m; 33°34'N, 132°56.2'E; 17 Jun. 2019; D. Kato leg. (BLKU) • 1♂; same data as previous except alt. 1387 m; 33°33.89'N, 132°56.1'E; L.-P. Kolcsár leg. (CKLP) • 1♀; same data as previous except alt. 890 m; 33°32.92'N, 132°56.88'E; L.-P. Kolcsár leg. (CKLP) • 2♂; Ehime, Kumakôgen-chô, Wakayama; alt. 930 m; 33°42.95'N, 132°6.5'E; 18 May 2019; L.-P. Kolcsár leg. (CKLP) • 1♂; Ehime, Matsuyama-shi, Jikibamachi; alt. 180 m; 33°51.69'N, 132°49.55'E; 16 May 2019; L.-P. Kolcsár leg. (CKLP) • 1♀; Ehime, Matsuyama-shi, Shukunomachi; alt. 240 m; 33°52.08'N, 132°50.09'E; 3 May 2019; L.-P. Kolcsár leg. (CKLP) • 1♀; Ehime, Matsuyama-shi, Yuyamayanagi; alt. 250 m; 33°53.41'N, 132°50.52'E; 3 May 2019; L.-P. Kolcsár leg. (CKLP) • 1♂, 1♀; Ehime, Saijô-shi, Nishinokawa-Tei, Mt Ishizuchi-san; alt. 1530 m; 33°45.29'N, 133°9.19'E; 16 Jun. 2019; D. Kato leg. (BLKU) • 1♂, 2♀; same data as previous except alt. 1480 m; 33°45.3'N, 133°9.23'E; 16 May 2019; L.-P. Kolcsár leg. (CKLP). [Kyushu] • 2♂, 2♀; Fukuoka, Fukuoka-shi, Jônan-ku, Katae, Mt Abura-yama; alt. 230 m; 33°31.83'N, 130°21.96'E; 19 Apr. 2014; D. Kato leg. (BLKU) • 1♂, 2♀; Fukuoka, Fukuoka-shi, Sawara-ku, Itaya, Mt Sefuri-san; alt. 970 m; 33°26.29'N, 130°22'E; 4 Jun. 2015; D. Kato leg. (BLKU) • 2♀; same data as previous except 10 Jun. 2015 • 2♀; same data as previous except 17 Jun. 2015 • 1♂; Fukuoka, Miyako-machi, Saigawa-Hobashira, Notȏge Pass; alt. 740 m; 33°29.74'N, 130°57.69'E; 12 May 2019; D. Kato leg. (BLKU) • 2♀; Fukuoka, Miyawaka-shi, Inunaki, Mt Inunaki-san; alt. 300 m; 33°40.87'N, 130°33.19'E; 5 May 2015; D. Kato leg. (BLKU) • 1♀; Saga, Kanzaki-shi, Sefuri-machi-Fukumaki; alt. 980 m; 33°26.04'N, 130°22.12'E; 23 May 2019; L.-P. Kolcsár leg. (CKLP) • 1♂; Saga, Saga-shi, Fuji-machi-Sekiya, Kase-gawa River near Hokuzan Dam; alt. 320 m; 33°25.99'N, 130°13.93'E; 23 May 2015; D. Kato leg. (BLKU) • 2♂; Miyazaki, Takachiho-chô, Gokasho, Mt Sobo-san, near Kitadani trailhead; alt. 1150 m; 32°49.36'N, 131°19.64'E; 22 May 2019; D. Kato leg. (BLKU).

#### Diagnosis.

Body blackish. Vertex sparsely pruinose, sometimes partly polished. Flagellomeres with basal 2–3 segments oval ventral sides with pubescences. Scutum sparsely pruinose or polished. Wing brownish tinged, with oval, dark-brown stigma and weakly dark seam on anterior part of cord. Halter yellowish. Interbase with outer lobe shorter than medial lobe in dorsal view; medial lobe medial to base of outer lobe about 2.3 times as long as wide. Aedeagus with rod-shaped distal part, 3.3–4.4 times as long as wide and almost straight.

#### Description.

**Male**. Body length 7.2–11.0 mm, wing length 7.1–11.9 mm.

***Head*:** subnitidous black, sparsely dusted with gray pruinosity; vertex with brighter gray pruinosity at anterior end; anterior part of vertex sometimes largely shiny except anterior end or with shiny and small, longitudinally long bacilliform area at middle posterior to anterior brighter area. Rostrum and mouthparts dark brown. Antenna with scape and pedicel dark brown; flagellum dusky yellow to brown; basal 2–3 flagellomeres oval, covered with pubescences ventrally.

***Thorax*:** subnitidous black, sparsely dusted with brownish pruinosity, sometimes polished and pruinosity absent on prescutum and most of scutal lobe; postpronotum yellowish or brownish. Wing (Fig. 2B) tinged with brown; basal and costal regions proximal to cord yellowish; stigma oval, dark brown, with faint outline; veins dark brown, yellowish on Sc and basal part of wing; weakly dark seam on fork of Rs to crossvein r-m. Halter yellow to dusky yellow. Legs mainly yellow to dusky yellow; fore coxa dark on basal half, sometimes entirely so, mid and hind coxae sometimes narrowly dark at bases; femora narrowly dark at tips; dark area on fore femur often extending to near middle; tibiae narrowly dark at tips; tarsi weakly dark from tip of tarsomere 1 to apical segment.

***Abdomen*:** subnitidous dark brown to black, sparsely covered with brownish pruinosity.

***Male terminalia*** (Fig. 2C–G): caudal margin of tergite 9 roundly produced at middle, with shallow U-shaped notch at center. Outer gonostylus in dorsal view (Fig. 2D) with tip narrowed and curved anteriorly, concaved margin with distinct teeth. Interbase with outer lobe shorter than medial lobe in dorsal view (Fig. 2E); outer lobe wide at base, inner basal end situated near middle of medial lobe (Fig. 2G); medial lobe strongly narrowed distally, weakly sinuous, distal part medial to base of outer lobe about 2.3 times as long as wide (Fig. 2G). Aedeagus with rod-shaped distal part, 3.3–4.4 times as long as wide and almost straight (Fig. 2E, F), usually shorter in specimens from southern part of Japan.

**Female**. Body length 7.0–11.7 mm, wing length 6.2–10.2 mm. Generally resembling male.

***Ovipositor*:** dark brown; yellow on cercus, hypogynial valve, and distal 1/4–1/3 of tergite 10; cercus 1.5 times as long as tergite 10.

#### Etymology.

The specific epithet is from the Latin *longus/longi* (long) + *penis* (penis) and refers to the long aedeagus of this species compared to the other Japanese species of the genus. The name is an adjective in nominative singular.

#### Distribution.

Japan (Hokkaido, Honshu, Shikoku, and Kyushu islands) (Fig. 5B).

#### Remarks.

This species is similar to *U. polytricha* Alexander, 1930 but is differentiated from it by the following characters: wing with stigma distinctly dark, fork of Rs to crossvein r-m weakly darkened (Fig. 2B) (stigma indistinct in *U. polytricha*; Fig. 4C); aedeagus with rod-shaped part more than three times as long as wide (Fig. 2F) (twice as long as wide in in *U. polytricha*; Fig. 4G). This species also resembles a Nearctic species, *U. nigronitida* Alexander, 1920, according to the original description (Alexander 1920b), but is distinguished from it by the following characters: antenna pale on flagellum, yellow to brown (black throughout in *U. nigronitida*); coxae with distal parts yellowish (coxae brownish black, hind pair paler in *U. nigronitida*); halter entirely yellowish (knob dark brownish black in *U. nigronitida*).

### Ulomorpha nigricolor

Taxon classificationAnimaliaDipteraLimoniidae

Alexander, 1924

5A28DC98-C7A2-5BB2-99CC-80712337C2EE

Figs 3 , 5A

Ulomorpha nigricolor Alexander 1924: 75 (type locality: Japan, Honshu I., Gunma or Fukushima, Lake Oze-numa); Alexander 1953a: 82; Alexander 1953b: 68; Nakamura 2014: 20; Oosterbroek 2020.

#### Material examined.

***Holotype*** ♂. Japan, Honshu, Lake Ozenuma, on boundary between Iwashiro-no-kuni and Kotsuke-no-kuni (between Fukuoka and Gunma); altitude 5460 feet; 36°55.62'N, 139°18.23'E (rough coordinate); 26 Jul. 1923; T. Esaki leg. (USNM).

***Non-types*.** Japan: [Honshu] • 1♀; Aomori, Nishimeya-mura; alt. 275 m; 40°31.39'N, 140°13.93'E; 23. Jun. 2012; D. Kato leg. (BLKU) • 1♂; same data as previous except 24. Jun. 2012 • 2♂, 1♀; Aomori, Nishimeya-mura, Kawaratai, Shirakami Nature Observation Garden; alt. 225 m; 40°31.13'N, 140°12.89'E; 21 Jun. 2013; D. Kato leg. (BLKU) • 1♂; Aomori, Nakadomari-machi, Ôsawanai, Ôsawanai Pond; alt. 35 m; 40°56.78'N, 140°27.74'E; 15 May 2014; D. Kato leg. (BLKU) • 1♂; same data as previous except 24 May 2014 • 1♂; Aomori, Towada-shi, Okuse, Tsutanuma Path; alt. 470 m; 40°35.45'N, 140°57.42'E; 23 May 2014; D. Kato leg. (BLKU) • 1♀; Iwate, Hachimantai-shi, near Tȏshichi Spa; alt. 1340 m; 39°56.55'N, 140°52.08'E; 28 Aug. 2014; D. Kato leg. (BLKU) • 1♂; Niigata, Echigo, Iwafune, Mt Zao; 38°4.64'N, 139°28.57'E (rough coordinate, altitude unknown); 3 May 1955; K. Baba leg. (USNM) • 1♀; Gifu, Mino, Sakauchi; 35°36.41'N, 136°22.96'E (rough coordinate, altitude unknown); 9 Jun. 1957; Mishima leg. (USNM) • 5♂, 4♀; Okayama, Maniwa-shi, Hiruzen-Shimotokuyama; alt. 780 m; 35°19.76'N, 133°35.84'E; 17 May 2015; D. Kato leg. (BLKU) • 2♂, 2♀; Okayama, Maniwa-shi, Hiruzen-Kamifukuda, Nawashirodani-gawa River; alt. 600 m; 35°19.19'N, 133°36.49'E; 29 Apr. 2016; D. Kato leg. (BLKU) • 1♂; Hiroshima, Akiôta-chô, Yokogô; alt. 890 m; 34°35.65'N, 132°8.7'E; 18 May 2015; D. Kato leg. (BLKU). [Shikoku] • 1♂; Ehime, Kumakôgen-chô, Nishidani, near Prefectural road 328; alt. 1430 m; 33°34'N, 132°56.2'E; 17 Jun. 2019; D. Kato leg. (BLKU) • 2♂, 2♀; same data as previous except alt. 1387 m; 33°33.89'N, 132°56.1'E; L.-P. Kolcsár leg. (CKLP) • 4♂; Ehime, Saijô-shi, Nishinokawa-Tei, Mt Ishizuchi-san; alt. 1530 m; 33°45.29'N, 133°9.19'E; 16 Jun. 2019; D. Kato leg. (BLKU) • 2♂; same data as previous except alt. 1480 m; 33°45.3'N, 133°9.23'E; 16 May 2019; L.-P. Kolcsár leg. (CKLP) • 1♂, same data as previous except 16 Jun. 2019. [Kyushu] • 3♂; Miyazaki, Takachiho-chô, Gokasho, Mt Sobo-san, near Kitadani trailhead; alt. 1150 m; 32°49.36'N, 131°19.64'E; 22 May 2019; D. Kato leg. (BLKU) • 5♂, 1♀; same data as previous except alt. 1182 m; 32°49.36'N, 131°19.64'E; L.-P. Kolcsár leg. (CKLP).

#### Diagnosis.

Body blackish. Vertex sparsely pruinose, anterior part largely polished. Flagellomeres oval on basal 2–4 segments; ventral sides with pubescences on basal 2–6 segments. Prescutum polished, with narrow longitudinal line of pruinosity at middle. Wing brownish tinged, with oval, dark-brown stigma and dark spot or seam each on Rs origin, anterior part of cord, crossvein m-cu, and outer end of cell d; dark spot at tip of A_2_ sometimes present. Halter yellowish. Interbase with outer lobe as long as medial lobe in dorsal view; medial lobe medial to base of outer lobe about 3.5 times as long as wide. Aedeagus with rod-shaped distal part, twice as long as wide and weakly curved ventrally.

#### Description.

**Male**. Body length 5.5–10.1 mm, wing length 5.3–10.0 mm.

***Head*:** subnitidous black, sparsely dusted with gray pruinosity; vertex with brighter gray pruinosity at anterior end; anterior part of vertex largely shiny except anterior end. Rostrum and mouthparts dark brown. Antenna with scape and pedicel dark brown; flagellum dusky yellow to dark brown; basal 2–4 flagellomeres oval; basal 2–6 segments covered with pubescences ventrally.

***Thorax*:** subnitidous black, sparsely dusted with brownish pruinosity; polished and pruinosity absent on prescutum and most of scutal lobe; postpronotum sometimes yellowish; prescutum with narrow longitudinal line of pruinosity at middle in whole length, sometimes anterior part of this line indistinct. Wing (Fig. 3B, C) tinged with brown; basal and costal regions proximal to cord yellowish; stigma oval, dark brown, faint in outline; veins dark brown, yellowish on Sc and basal part of wing; dark, wide seam on fork of Rs to crossvein r-m; narrow, dark seam on each of crossvein m-cu and outer end of cell d, but one on latter sometimes indistinct; dark small spot at Rs origin and sometimes with additional one at tip of A_2_ (Fig. 3C), this anal spot usually present in specimens from southern part of Japan. Halter yellow to pale yellow. Legs mainly yellow to dusky yellow; fore coxa dark on basal 1/2, sometimes entirely so; mid and hind coxae yellow to dark brown, sometimes dark at bases in case of yellowish coxae; femora narrowly dark at tips, dark area on fore femur sometimes occupying distal 1/3; tibiae narrowly dark at tips; tarsi weakly dark from tip of tarsomere 1 to apical segment.

***Abdomen*:** subnitidous dark brown to black, sparsely covered with brownish pruinosity.

***Male terminalia*** (Fig. 3D–H): caudal margin of tergite 9 almost straight or weakly convex at middle, with shallow U-shaped notch at center. Outer gonostylus in dorsal view (Fig. 3E) relatively wide on distal part, tip narrowed and curved anteriorly, concaved margin with indistinct teeth. Interbase with outer lobe as long as medial lobe in dorsal view (Fig. 3F); outer lobe wide at base, inner basal end situated near middle of medial lobe (Fig. 3H); medial lobe strongly narrowed and rod-shaped on distal half, distal part medial to base of outer lobe about 3.5 times as long as wide (Fig. 3H); Aedeagus with rod-shaped distal part, twice as long as wide and tip weakly curved ventrally (Fig. 3G).

**Female**. Body length 6.3–9.6 mm, wing length 5.4–8.2 mm. Generally resembling male.

***Ovipositor*** (Fig. 3I): dark brown; yellow on cercus, hypogynial valve, and distal 1/4–1/3 of tergite 10; cercus 2–2.5 times as long as tergite 10.

#### Distribution.

Japan (Hokkaido, Honshu, Shikoku, and Kyushu islands) (Fig. 5A) and North Korea.

#### Remarks.

This species is easily distinguished from the other species of the genus by the following combination of characters: thorax and abdomen excluding legs dark brown to black; wing with distinct dark areas each at origin of Rs, on fork of Rs to crossvein r-m, and outer end of cell d (Fig. 3B, C).

### Ulomorpha polytricha

Taxon classificationAnimaliaDipteraLimoniidae

Alexander, 1930

41D33DDB-EFF0-5048-8E1C-DEDC10FAAB96

Figs 4 , 5A

Ulomorpha polytricha Alexander 1930a: 72 (type locality: Japan, Nansei Islands, Yaku-shima I., Kosugidani); Alexander 1930b: 508; Nakamura 2014: 20; Oosterbroek 2020.

#### Material examined.

***Holotype*.** ♂, Japan, Nansei Islands, Yaku-shima I., Kosugidani; altitude 2,500 feet; 30°20.84'N, 130°35.25'E (rough coordinate); 29 Apr. 1929; S. Issiki leg. (USNM).

***Non-types*.** Japan: [Nansei Islands] Yakushima I.: • 17♂, 1♀; near Shirataniunsui-kyô Valley; alt. 600 m; 30°23.04'N, 130°34.37'E; 25 Apr. 2019; D. Kato leg. (BLKU) • 1♂; same data as previous except 27 Apr. 2019 • 1♂; unnamed stream near Kigensugi; alt. 1270 m; 30°18.1'N, 130°32.56'E; 25 Apr. 2019; D. Kato leg. (BLKU) • 2♂, 1♀; unnamed stream near Mt Mae-dake; alt. 625 m; 30°18.91'N, 130°36.61'E; 25 Apr. 2019; D. Kato leg. (BLKU).

#### Diagnosis.

Body blackish. Vertex sparsely pruinose. Basal 2–3 flagellomeres oval, with pubescence ventrally. Prescutum sparsely pruinose. Wing brownish tinged, unpatterned except indistinctly darker stigma. Halter yellow. Interbase with outer lobe shorter than medial lobe in dorsal view, medial lobe medial to base of outer lobe more than twice as long as wide. Aedeagus with rod-shaped distal part, about 2.2 times as long as wide and almost straight.

#### Description.

**Male**. Body length 5.7–8.0 mm, wing length 6.0–8.1 mm.

***Head*:** subnitidous black, sparsely dusted with gray pruinosity; vertex with brighter gray pruinosity at anterior end. Rostrum and mouthparts dark brown. Antenna brown; scape and pedicel sometimes slightly darker; basal 2–3 flagellomeres oval, ventrally with pubescence (Fig. 4B).

***Thorax*:** subnitidous dark brown to black, sparsely dusted with brownish pruinosity; postpronotum yellowish or brownish. Wing (Fig. 4C) tinged with brown;, basal region yellowish; stigma indistinctly darker; veins dark brown, yellowish on basal part of wing; barely dark seam sometimes present on fork of Rs to crossvein r-m. Halter yellow. Legs mainly yellow to dusky yellow; fore coxa dark on basal half; femora narrowly dark at tips of mid and hind pairs; fore femur dark on distal 2/3; tibiae narrowly dark at tips; tarsi weakly dark from tip of tarsomere 1 to apical segment.

***Abdomen*:** subnitidous dark brown to black, sparsely covered with brownish pruinosity.

***Male terminalia*** (Fig. 4D–H): caudal margin of tergite 9 roundly produced at middle, with shallow U-shaped notch at center. Outer gonostylus in dorsal view with tip narrowed and strongly curved anteriorly, concaved margin with small teeth (Fig. 4E). Interbase with outer lobe shorter than medial lobe in dorsal view (Fig. 4F); outer lobe weakly wide at base, inner basal end at basal 1/3 of medial lobe (Fig. 4H); medial lobe gradually narrowed distally, distal part medial to base of outer lobe more than twice as long as wide (Fig. 4H). Aedeagus with rod-shaped distal part, about 2.2 times as long as wide and almost straight (Fig. 4G).

**Female**. Body length 7.2–7.6 mm, wing length 6.7–7.1 mm. Generally resembling male.

***Ovipositor*:** dark brown; yellow on cercus, hypogynial valve, and distal 1/4 of tergite 10; cercus twice as long as tergite 10.

#### Distribution.

Japan (Nansei Islands: Yakushima Island) (Fig. 5A).

#### Remarks.

This species is similar to *U. amamiana* sp. nov. See the key to the Japanese species above for differentiation and diagnostic characters.

## Supplementary Material

XML Treatment for Ulomorpha

XML Treatment for Ulomorpha amamiana

XML Treatment for Ulomorpha longipenis

XML Treatment for Ulomorpha nigricolor

XML Treatment for Ulomorpha polytricha

## References

[B1] Alexander CP (1919) The crane-flies of New York. Part I. Distribution and taxonomy of the adult flies. Memoirs, Cornell University Agricultural Experiment Station 25: 767–993.

[B2] Alexander CP (1920a) The crane-flies of New York. Part II. Biology and phylogeny. Memoirs, Cornell University Agricultural Experiment Station 38: 691–1133. 10.5962/bhl.title.33641

[B3] Alexander CP (1920b) Undescribed Tipulidae (Diptera) from western North America. Proceedings of the California Academy of Sciences (Series 4) 10(5): 35–46.

[B4] Alexander CP (1924) New or little-known Tipulidae (Diptera). XXVI. Palaearctic species. Annals and Magazine of Natural History (Series 9) 15(9): 65–81. 10.1080/00222932508633181

[B5] Alexander CP (1930a) New or little-known Tipulidae from eastern Asia (Diptera). VI. Philippine Journal of Science 42: 59–83.

[B6] Alexander CP (1930b) New or little-known Tipulidae from eastern Asia (Diptera). VII. Philippine Journal of Science 42(4): 507–535.

[B7] Alexander CP (1936) New or little-known Tipulidae from eastern Asia (Diptera). XXX. Philippine Journal of Science 60: 165–204.

[B8] Alexander CP (1953a) The insect fauna of Mt. Ishizuchi and Omogo Valley, Iyo, Japan. The Tipulidae (Diptera). Transactions of the Shikoku Entomological Society 3: 71–83

[B9] Alexander CP (1953b) Records and descriptions of Japanese Tipulidae (Diptera). Part I. The crane-flies of Shikoku. I. Philippine Journal of Science 82: 21–75.

[B10] Alexander CP McAtee WL (1920) Diptera of the superfamily Tipuloidea found in the District of Columbia. Proceedings of the United States National Museum 58: 385–435. 10.5479/si.00963801.58-2344.385

[B11] Bergroth EE (1891) Beitrag zur Tipuliden-Fauna der Schweiz. Mitteilungen der Naturforschenden Gesellschaft in Bern 1890: 129–138.

[B12] Kato D (2020) New records of Japanese Limoniinae (Diptera: Limoniidae). Makunagi/Acta Dipterologica 31: 15–52.

[B13] Kolcsár L-P Kato D Gamboa M Watanabe K (2020) [in press] Revision of Japanese species of *Nipponomyia* Alexander, 1924 (Diptera, Pediciidae). ZooKeys. 10.3897/zookeys.1000.55021 PMC7728730 33354136

[B14] Komaki S Igawa T (2017) The widespread misconception about the Japanese major biogeographic boundary, the Watase line (Tokara gap), revealed by bibliographic and beta diversity analyses. bioRχiv: e186775. 10.1101/186775

[B15] Latreille PA (1809) Genera crustaceorum et insectorum secumdum ordinem naturalem in familias disposita, iconibus exemplisque plurimis explicate. Vol 4. Amand Koenig, Paris and Strasbourg, 399 pp.

[B16] McAlpine JF (1981) Morphology and terminology: Adults. In: McAlpine JF Petersen BV Shewell GE Teskey HJ Vockeroth JR Wood DM (Eds) Manual of Nearctic Diptera 1.Biosystematic Research Institute, Ottawa, 1–63.

[B17] Merz B Haenni J-P (2000) Morphology and terminology of adult Diptera (other than terminalia). In: Papp L Darvas B (Eds) Contributions to a Manual of Palaearctic Diptera 1.Science Herald, 21–51.

[B18] Nakamura T (2014) Family Limoniidae. In: Nakamura T Saigusa T Suwa M (Eds) Catalogue of the Insects of Japan.Volume 8 Diptera (Part 1 Nematocera – BrachyceraAschiza). Touka Shobo, Fukuoka, 9–53.

[B19] Oosterbroek P (2020) Catalogue of the Craneflies of the World (Diptera, Tipuloidea: Pediciidae, Limoniidae, Cylindrotomidae, Tipulidae). https://ccw.naturalis.nl/index.php [accessed on 2020-03-30]

[B20] Oosterbroek P van Theowald L (1991) Phylogeny of the Tipuloidea based on characters of larvae and pupae (Diptera, Nematocera) with an index to the literature except Tipulidae. Tijdschrift voor Entomologie 134: 211–267.

[B21] Osozawa S Shinjo R Armid A Watanabe Y Horiguchi T Wakabayashi J (2012) Palaeogeographic reconstruction of the 1.55 Ma synchronous isolation of the Ryukyu Islands, Japan, and Taiwan and inflow of the Kuroshio warm current. International Geology Review 54: 1369–1388. 10.1080/00206814.2011.639954

[B22] Osten Sacken CR (1860) New genera and species of North American Tipulidae with short palpi, with an attempt at a new classification of the tribe. Proceedings of the Academy of Natural Sciences of Philadelphia 1859: 197–254.

[B23] Osten Sacken CR (1869) Monographs of the Diptera of North America. Part IV. Smithsonian Miscellaneous Collections 8(219): 1–345.

[B24] Ribeiro GC (2008) Phylogeny of the Limnophilinae (Limoniidae) and early evolution of the Tipulomorpha (Diptera). Invertebrate Systematics 22: 627–694. 10.1071/IS08017

[B25] Starý J (2008) The wing stalk in Diptera, with some notes on the higher-level phylogeny of the order. European Journal of Entomology 105: 27–33. 10.14411/eje.2008.003

[B26] Sintenis F (1889) Uber *Limnophila pilicornis* Zett. Sitzungsberichte der Naturforscher-Gesellschaft bei der Universitat Dorpat 8: 396–398.

